# Percutaneous or Side-Arm Graft Right Subclavian Artery Cannulation via Median Sternotomy

**DOI:** 10.1055/s-0039-3401019

**Published:** 2020-02-04

**Authors:** Corrado Cavozza, Antonio Campanella, Pellegrino Pasquale, Andrea Audo

**Affiliations:** 1 Department of Cardiothoracic and Vascular Surgery, Cardiac Surgery, Santissimi. Antonio e Biagio e Cesare Arrigo Hospital, Alessandria, Italy

**Keywords:** aortic dissection, cannulation, right subclavian artery cannulation

## Abstract

Several cannulation sites alternative to the ascending aorta, such as femoral, right axillary, carotid, innominate artery, and, less commonly, apical sites, have been proposed. Cannulation of the right subclavian artery, through sternotomy, is one possible means of establishing cardiopulmonary bypass, hence avoiding a second surgical incision. In our experience, cardiopulmonary bypass flow was adequate and circulatory arrest with antegrade cerebral perfusion was successfully performed in all cases. There was no in-hospital mortality.

## Introduction

When cannulation of the ascending aorta is not advisable, such as in cases of Type-A aortic dissection, alternatives including femoral, right axillary, carotid, and, less commonly, apex of the left ventricle have been proposed. 1 2 3 4

Cannulation of the femoral vessels carries risk of poor perfusion and retrograde thrombotic embolism. 5 The cardiac apex can be very fragile. Surgical approach to right axillary artery may be time consuming, especially in obese patients, and this should be weighed against the urgency of the operation. In addition, injury to the brachial plexus, arm hyperperfusion or seroma formation, although rare in experienced hands, are potential complications. Carotid artery is often affected by dissection. Use of the innominate artery, provided that it is not dissected, offers advantages similar to those of the right axillary artery, while eliminating some of the concerns over the added length of the procedure. 1 4 When the innominate artery is not suitable for arterial cannulation, the right subclavian artery, uncommonly affected by the acute dissection process, may be cannulated either directly or by sewing an end-to-side 8-mm polyester graft to the artery. This can permit systemic flow with antegrade perfusion without creating any other surgical incision than sternotomy.

## Techniques and Results

**Fig. 1:**
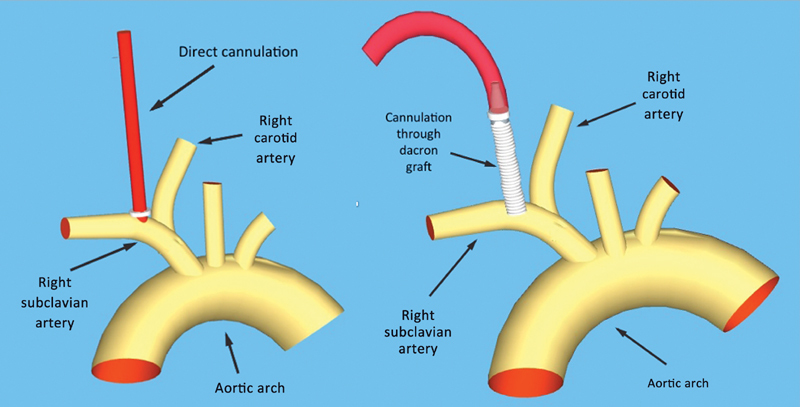
Schematic representation of percutaneous or through graft subclavian artery cannulation.

**Fig. 2:**
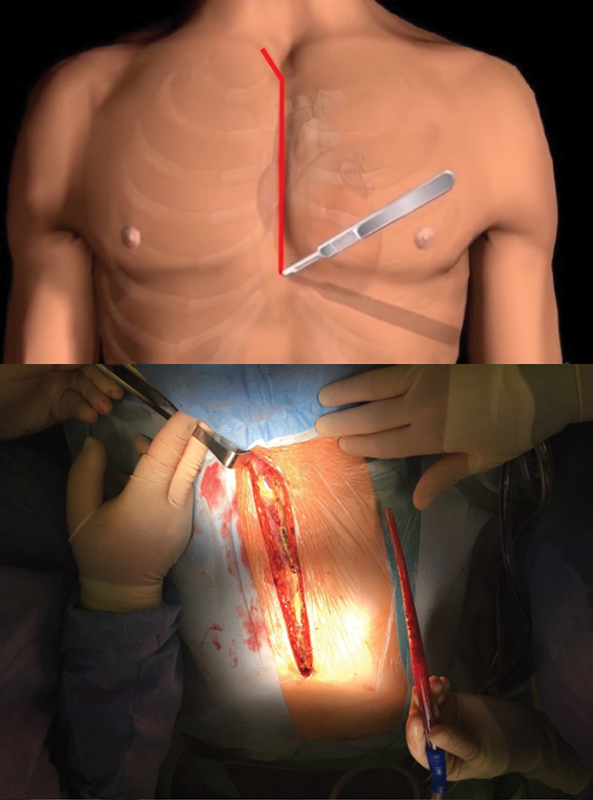
Sternotomy incision with neck extension.

**Fig. 3:**
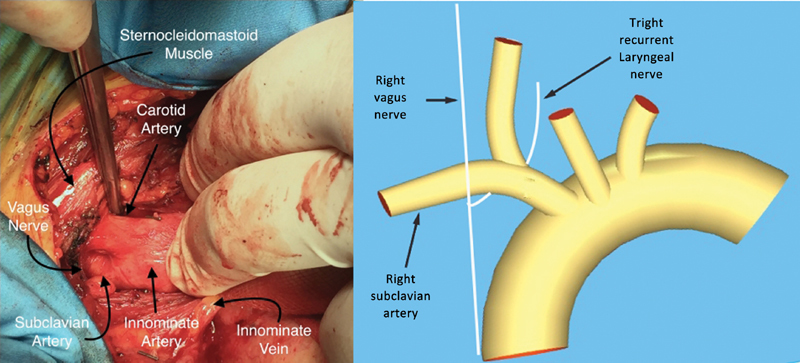
The vagus nerve then crosses anterior to the first part of the subclavian artery at the lower margin and gives off its right recurrent laryngeal branch surrounding the artery.

**Fig. 4:**
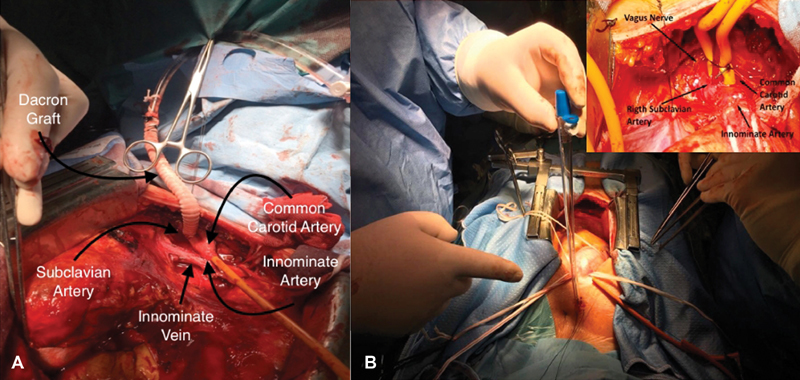
( **A** ) Rigth subclavian artery cannulation trough side graft. ( **B** ) Percutaneous right subclavian artery cannulation.

Twenty consecutive patients with Type A aortic dissection received cannulation of the right subclavian artery, directly through percutaneous cannula placement or using a dacron graft, after sternotomy, before pericardial incision ( Fig. 1 ; Table 1 ). Blood pressure in both arms and core temperature were invasively monitored. Median sternotomy is extended to the right neck ( Fig. 2 ), the innominate vein is identified and encircled with an umbilical tape, allowing the vein to be retracted inferiorly. The innominate artery is exposed, and dissected up to the bifurcation and subclavian artery. The subclavian artery is identified and dissected. The first part of the right subclavian artery passes from the bifurcation of the right brachiocephalic trunk deep to the right sternocostal joint to the medial edge of the scalenus anterior muscle and is crossed by the vagus nerve near brachiocephalic artery division. Exposure is facilitated with a retractor like farabeuf. The right vagus descends within the carotid sheath between the internal jugular vein and the internal and common carotid artery, then crosses anterior to the first part of the subclavian artery at the lower margin and gives off its right recurrent laryngeal branch surrounding the artery ( Fig. 3 ). The nerve receives close attention from surgeons because the nerve is at risk for injury that may prevent with appropriate surgical exposure. After heparinization, the subclavian artery can be cannulated directly percutaneous if transverse diameter is suitable distally to the innominate artery bifurcation. A 20 or 18 French OptiSiteTM arterial cannula (Edwards Lifesciences, Irvine, CA) via Seldinger technique or through a skin incision a 22 French DLP Flexible Arch Arterial Cannula (Medtronic, Inc., Minneapolis, MN) is inserted. Afterwards longitudinal arteriotomy is performed and an 8-mm dacron graft anastomosed, after partially vascular clamp occlusion, taken care not to damage vagus nerve that was easily moved away ( Fig. 4 ). The arterial line can be and venous cannulation was performed as appropriate. An adequate pump flow rate was achieved. No instances of high pressure in the arterial line were observed. Core temperature was decreased to 26°C and after stopping systemic perfusion, unilateral antegrade cerebral perfusion was performed at 10 mL/kg, via the subclavian artery. To assess brain perfusion, we used solvent infrared spectroscopy capillary saturation by a dual sensor for NIRS (near infrared spectroscopy). Eight patients underwent bilateral perfusion because of unilateral drop in saturation to less than 20% of the baseline value of NIRS. Right radial artery blood pressure was maintained between 50 and 60 mm Hg. Mean cardiopulmonary bypass time was 183 ± 82 minutes. Mean circulatory arrest time was 38.6 ± 15 minutes. There were no cases of traumatic dissection of the subclavian artery. There were no right arm vascular complications and in-hospital mortality. Three patients showed temporary neurological dysfunction defined as presence of reversible postoperative motor deficit, confusion, agitation, or transient delirium. The brain computed tomography scan was normal and all symptoms were resolved before discharge. One patient presented with a non-Q wave acute myocardial infarction (patient with preoperative Type-A aortic dissection and occlusion of the right coronary). Two patients developed a temporary disturbance in renal function but recovered completely.

**Table 1:** Patients characteristics

Variables	Value
No.	20
Mean age in y (range)	74 ± 7.5
Male	12
Preoperative shock	5
Preoperative CPA	1
*Organ malperfusion* :	
Cerebral	4
Coronary	3
Kidney	7
Spinal cord	5
Leg	6
Aortic regurgitation	18
*Location of primary tear:*	
Aortic arch	2
Ascending aorta	18
Cardiopulmonary bypass time (min)	183 ± 82
Circulatory arrest time (min)	38.6 ± 15
Lowest tympanic temperature (°C)	24.6 ± 1.3
*Concomitant procedures* :	
Coronary artery bypass grafting	2
Aortic valve replacement	5
Root replacement	3
Conversion to TAR	4
Reexploration for surgical bleeding	3
Major adverse events	
Hospital death	0
Stroke	2
Paraplegia	1
Respiratory failure	2
Permanent hemodialysis	1

Abbreviations: CPA, perioperative cardiac arrest; CPB, cardiopulmonary bypass; TAR, total arch replacement.

Note: respiratory failure means postoperative pneumonia, pneumothorax, and tracheotomy.

## Discussion

Nowadays the subclavian artery is our preferred site for cannulation when dissection involves the innominate artery, thus avoiding the complications of a second surgical incision. Dissection is still the bigger issue, but the subclavian artery usually is not involved in the dissection process, although is generally more fragile than the femoral artery and cannulation can therefore be traumatic. 6 7 In the present series, there were no vascular complications arising from the cannulation of the subclavian artery. Systemic perfusion was in all cases controlled without incident. Closure of the arteriotomy was quick and easy.
